# “Effect of a support program on fathers’ stress and anxiety during initial NICU encounter with premature infants: a randomized clinical trial”

**DOI:** 10.1186/s12978-025-02204-w

**Published:** 2025-12-29

**Authors:** Seyedeh Batool Hasanpoor-Azghady, Seyedeh Saeedeh Mousavi, Seyedeh Pariya Jafari, Fatemeh Razavinia, Shima Haghani

**Affiliations:** 1 Department of Midwifery and Reproductive, Nursing and Midwifery Care Research Center, Health Management Research Institute, School of Nursing and Midwifery, Iran University of Medical Sciences, Tehran, Iran; 2 School of Nursing and Midwifery, Iran University of Medical Sciences, Tehran, Iran; 3 Department of Midwifery and Reproductive, School of Nursing and Midwifery, Iran University of Medical Sciences, Tehran, Iran; 4 Sexual and Reproductive Health Research Center, Mazandaran University of Medical Sciences, Sari, Iran; 5 Nursing Care Research Center, Iran University of Medical Sciences, Tehran, Iran

**Keywords:** Premature baby, Stress, Anxiety, Support program, NICU

## Abstract

**Background:**

The birth of a preterm baby and subsequent hospitalization in the neonatal intensive care unit (NICU) pose significant emotional challenges for fathers. Fathers often suppress their feelings to fulfill a supportive role, but active participation in caregiving may alleviate their stress and enhance bonding. This study aimed to evaluate the impact of a support program on the stress and anxiety of fathers during their first encounter with a preterm baby.

**Methods:**

This randomized clinical trial (RCT) was conducted with a 1:1 allocation ratio at Shahid Motahari Hospital, Urmia, Iran. Eligible fathers (*n* = 80) of preterm babies were randomly assigned to either an experimental (*n* = 40) or a control (*n* = 40) group using a random block method with block sizes of four. Due to dropouts, 37 fathers in the experimental group and 36 in the control group were included in the analysis. The experimental group received a support program, including individualized informational and emotional support, in addition to standard NICU support. The control group received only standard NICU support. Anxiety was measured using the State-Trait Anxiety Inventory (Only the state anxiety subscale), before the first encounter and three days post-birth. Stress was assessed on day three using the Parental Stressor Scale: NICU (PSS: NICU).

**Results:**

Post-intervention, the experimental group exhibited significantly lower mean PSS: NICU scores (M = 64.32, SD = 24.12) compared to the control group (M = 109.75, SD = 22.24; mean difference=-45.43, 95% CI [-34.58, -56.26], *p* < 0.001). Among the subscales of stress, the parent-child relationship and parenting roles, despite a significant decrease after the intervention in the experimental group, still had the highest stress for fathers with preterm babies. The mean State anxiety score in the experimental group (M = 43.21, SD = 9.68) was significantly lower than in the control group (M = 54.22, SD = 10.39; mean difference= -11.01, 95% CI [-6.31, -15.69], *p* < 0.001), indicating reduced anxiety.

**Conclusion:**

The structured support program significantly reduced stress and anxiety among fathers of preterm babies. These findings suggest that health policymakers should consider integrating such interventions into NICU care protocols to support paternal well-being and family-centered care.

**Trial registration:**

IRCT20200120046200N1. Dated 11/5/2020 prospectively registered. (https://irct.behdasht.gov.ir/user/trial/46722/view).

**Supplementary Information:**

The online version contains supplementary material available at 10.1186/s12978-025-02204-w.

## Introduction

A preterm birth, defined as delivery before 37 weeks of gestation with a birth weight below 2500 g, remains a significant global health challenge. Despite extensive research, effective strategies to prevent preterm birth are still limited [1, 2]. According to the World Health Organization, around 13.4 million babies were born preterm worldwide in 2020, representing roughly 10% of all live births, with rates ranging from 5 to 7% in Europe to 10–15% in low- and middle-income countries [2, 3]. These figures indicate a slight increase compared with earlier estimates, such as 12.8% in 2006 and 27% in 2017, reported by the International Center for Health Statistics [2–4].

The birth of a preterm infant and subsequent hospitalization in the neonatal intensive care unit (NICU) is a profoundly stressful experience for parents [5, 6]. The NICU environment, characterized by continuous noise, medical equipment, and invasive procedures, exacerbates parental distress [7–10]. During the first week of hospitalization, parents often report feelings of shock, anxiety, and emotional overwhelm [11, 12].

While most studies have focused on mothers and infants, fathers’ experiences have been underexplored [13–15]. Fathers of preterm infants face unique pressures, balancing their role as a supporter for the mother and infant while grappling with heightened stress and disrupted bonding due to separation and the NICU’s clinical setting [16, 17]. These stressors may hinder fathers’ acceptance of their parental role, affect their emotional well-being, and disrupt parent-infant interactions, potentially influencing overall family dynamics [6].

Existing literature on parental support in the NICU predominantly targets mothers or employs group-based interventions, such as psychological training, relaxation techniques, or educational sessions [6, 18]. However, studies specifically addressing fathers’ needs are scarce and yield conflicting results. For instance, Matricardi et al. found that joint observation and infant massage by parents did not significantly reduce fathers’ stress in the first week post-birth [19]. Similarly, Turan et al. reported that NICU tours and group-based educational interventions had a limited impact on fathers’ stress levels [20]. In contrast, Shahkolahi et al. reported that targeted informational and psychological group sessions conducted 2–4 days after birth significantly reduced fathers’ stress [21]. These inconsistencies highlight a critical research gap: the lack of individualized interventions tailored to fathers, particularly during the critical “first encounter” with their preterm infant. Given mothers’ post-birth recovery needs, fathers’ active participation in early infant care is vital yet understudied [19–21].

No prior studies have specifically investigated interventions targeting fathers’ stress and anxiety during the initial encounter with a preterm infant [19–21]. This gap highlights the need for a tailored support program, delivered at a critical time, to improve paternal well-being and promote family-centered care. Therefore, this study aims to evaluate the impact of an individualized support program on the stress and anxiety of fathers during their first encounter with a preterm infant in the NICU.

## Method

### Study design

This randomized clinical trial (RCT) was conducted with a 1:1 allocation ratio at Shahid Motahari Hospital, Urmia, Iran, from November 2021 to May 2022. The study population included fathers of preterm infants (born before 37 weeks of gestation, with a birth weight < 2500 g) admitted to the neonatal intensive care unit (NICU). The study adhered to the Consolidated Standards of Reporting Trials (CONSORT) guidelines. Ethical approval was obtained from the Medical Research Ethics Committee of the Iran University of Medical Sciences (Code IR.IUMS.REC.1398.1361), and all participants provided written informed consent. The trial was registered at the Iranian Registry of Clinical Trials (IRCT20200120046200N1). Due to the nature of the intervention (active support program versus standard care), blinding of participants and NICU staff was not feasible; however, outcome assessors (data collectors administering questionnaires) were blinded to group allocation to reduce assessment bias.

### Sample size Estimation

A power analysis was performed with an alpha of 0.05 and a beta of 0.2 (80% power). Based on a previous study [22], a 9-point difference in Parental Stressor Scale: NICU (PSS: NICU) scores between groups was anticipated, resulting in a required sample size of 35 participants per group. To account for a 15% dropout rate, 40 fathers were recruited per group (*N* = 80). After accounting for dropouts, 37 fathers in the experimental group and 36 in the control group were included in the analysis.

### Participants

Inclusion criteria for fathers were: (a) age ≥ 18 years, (b) having literacy, (c) ability to communicate verbally, and (d) entering the study within 12 h after the birth of a premature baby. Inclusion criteria for premature infants included (a) gestational age < 37 weeks, (b) fifth-minute Apgar score > 5 and (c) singleton birth.

Exclusion criteria for fathers included (a) employment in health centers, (b) stressful events in the past 6 months (e.g., divorce, job loss), (c) substance abuse, (d use of medications for mental disorders, (e) presence or history of mental illness as reported by the research unit, and (f) history of infertility. Exclusion criteria for premature infants included (a) congenital malformation or any serious debilitating condition, such as grade 3 or 4 intraventricular hemorrhage. Withdrawal from the study was limited to cases of infant death.

### Randomization

Randomization and allocation concealment were performed using https://www.randomization.com. An independent researcher entered the number of groups [2], block size [4], and total participants (80) into the platform. Based on the generated randomization sequence, this researcher prepared 80 sealed envelopes containing group assignments (Group A: experimental; Group B: control). Envelopes were opened sequentially to assign participants, ensuring proper allocation concealment.

### Measures

Data were collected using three instruments:

1.  Demographic information form: This form included parental age, education and occupation, economic status, insurance status and its type, number of living children (including the current birth), type of mother’s last birth, infant’s sex, age at birth, Apgar score, and birth weight.
2.  Parental Stressor Scale: NICU (PSS: NICU): Developed by Miles et al. in 1993 and revised in 1998, 2002, and 2011. This self-report questionnaire assesses the stress levels of parents with premature infants hospitalized in the NICU. It comprises 26 items across three subscales: neonatal intensive care unit environment (5 items), the appearance and behavior of the baby and the special treatments that are given to the baby (14 items), and Parent-child relationship and parenting roles (7 items). Responses are rated on a six-point Likert scale: “I have not encountered this in the department”, “No stress”, “It causes a little stress”, “It causes moderate stress”, “It causes a lot of stress”, and it creates too much stress. Items marked as “I did not encounter this item” are treated as missing data. Stress scores for each item range from 0 to 4, with higher scores indicating greater parental stress. Total scores are categorized into five levels: very low stress (≤ 20), low stress (21–40), moderate stress (41–60), high stress (61–80), and very high stress (≥ 81) [23]. Cronbach’s alpha of the Persian version was calculated for the total questionnaire (0.87) and three subscales of the questionnaire, respectively: the neonatal intensive care unit environment (0.77), the appearance and behavior of the baby and the special treatments that are given to the baby (0.77), and Parent-child relationship and parenting roles (0.86) [24, 25].
3.  State-Trait Anxiety Inventory (STAI): Developed by Spielberger in 1970 and revised in 1983. The STAI is a self-report instrument with two subscales, each composed of 20 items. The state anxiety subscale assesses how participants feel “at this moment”, while the trait anxiety subscale measures how the participant “generally feels”. Since trait anxiety reflects a stable predisposition, which typically requires long-term psychosocial intervention to change, we only measured state anxiety in this study. Response options range from one (not at all) to four (very much so) for the state subscale. Higher scores indicate greater anxiety. Total scores ranging from 20 (not anxious) to 80 (very anxious) [26]. This scale has concurrent validity of 0.73 to 0.85 with other anxiety questionnaires. The reliability (*r* = 0.97) of the Persian version of the questionnaire has been confirmed using a test and retest [27].

### Experimental group

Eligible fathers were directed to a designated hospital room, where they completed the demographic form and State Anxiety Inventory (baseline assessment). Following randomization, participants in the experimental group received a single-session intervention adapted from study of Mousavi et al. [28]. The intervention consisted of three components: [1] Visual preparation and hope enhancement: Fathers viewed two photo albums, one illustrating the developmental progression of a preterm infant from birth to approximately two years of age, and another showing healthy children born preterm at various birth weights and ages— to help them become familiar with their infant’s appearance and foster a sense of hope [2]. Cognitive–emotional reinforcement: Fathers were encouraged to strengthen their beliefs in trust, patience, and resilience; and [3] Practical engagement training: Fathers were trained on proper storage and transfer of expressed breast milk to enhance their involvement in infant care. Afterwards, fathers were escorted to the NICU, introduced to the staff, and guided to their infant’s bedside. They were encouraged to engage in gentle, comforting interactions (e.g., continuous soft touch, whispering, eye contact). NICU staff explained the preterm infant’s features, NICU equipment, and routine procedures in simple terms, addressing all addressing all fathers’ questions.

Fathers also received an illustrated booklet and DVD containing educational material about preterm infant care, alternative feeding methods, and common neonatal issues, which they were asked to review over three days. A contact number was provided for follow-up questions. On the third day, fathers completed the State Anxiety Inventory (second time) and the PSS: NICU (first time).

### Control group

The control group (*n* = 40; analyzed *n* = 36) received standard NICU support, limited to routine explanations about their infant’s condition by department nurses, without any additional intervention during the study period. Fathers completed the demographic form, the State Anxiety Inventory (baseline and day three), and PSS: NICU (day three), following the same schedule as the experimental group. For ethical considerations, after completing the questionnaires on day three, fathers in control group received the same educational booklet, DVD, and follow-up contact number as those in the intervention group.

#### Data analysis

Data were analyzed using SPSS software version 25.0. Demographic characteristics were summarized as frequencies and percentages for categorical variables, and as means ± standard deviations for continuous variables. The normal distribution of the data was first examined using the Kolmogorov-Smirnov test. Baseline characteristics were compared between groups using Chi-square or Fisher’s exact tests for categorical variables and independent t-tests for continuous variables. Post-intervention anxiety and PSS: NICU scores were compared between groups using independent t-tests. Missing data, resulting from dropouts (7 in total, < 10% of the sample), were handled using complete-case analysis, as missingness was assumed to be at random based on no significant differences in baseline characteristics between completers and non-completers (*p* > 0.05, assessed via t-tests and Chi-square tests). All statistical tests were two-tailed, with statistical significance set at *p* < 0.05.

## Results

**Fig. 1:**
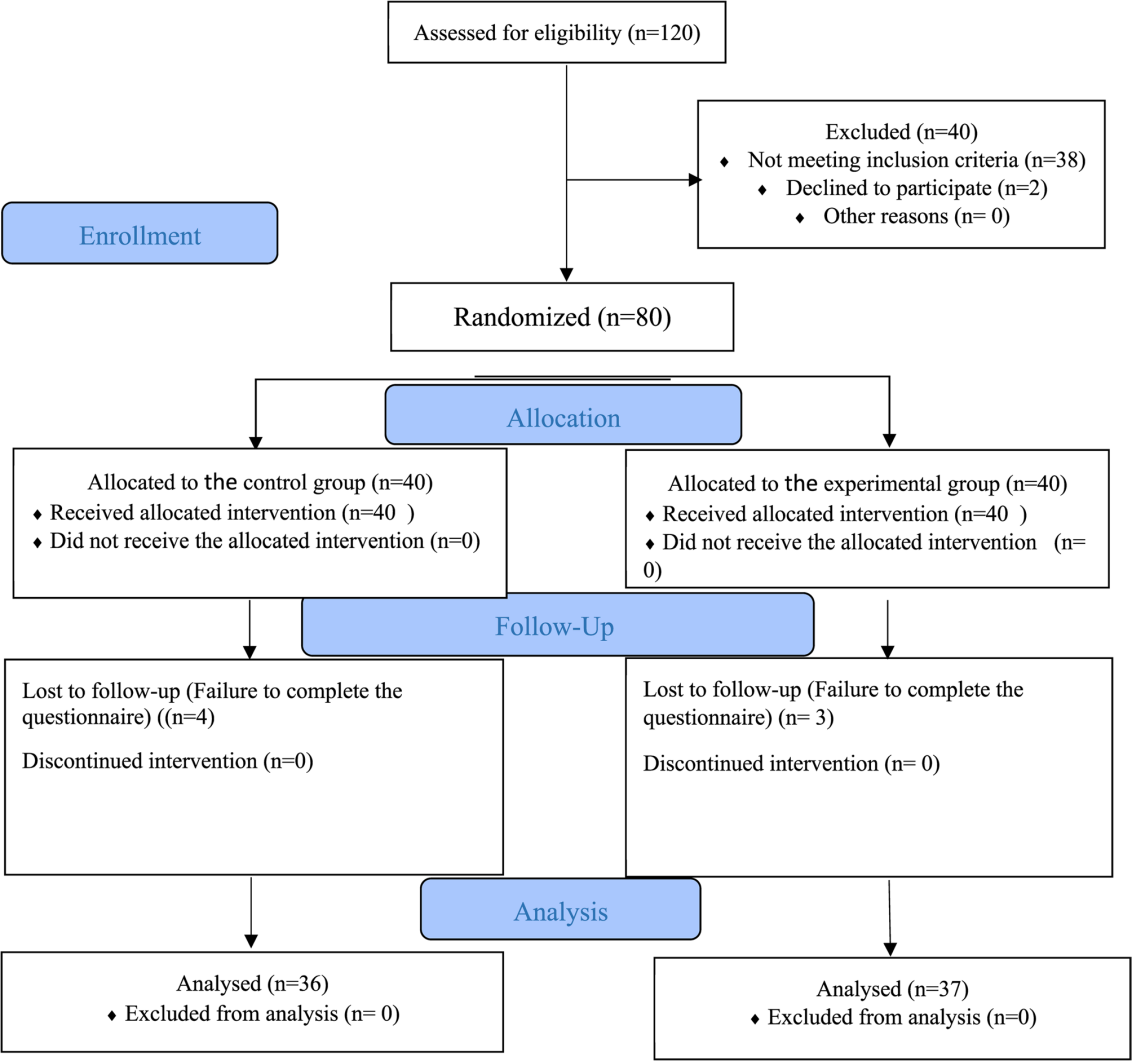
The study flowchart

Of 40 participants initially enrolled in each group, four people from the control group and three people from the experimental group were excluded due to failure to complete the questionnaire. Consequently, the control group consisted of 36 participants and the experimental group of 37 participants (Fig. 1).

There was no significant difference between the two groups in terms of parents of premature babies (age of mother and father, education of mother and father, occupation of mother and father, having insurance, economic status and number of children). Although the primary data were collected from fathers, maternal background characteristics are also presented to provide a more comprehensive demographic profile of the participating families (Table 1).

**Table 1:** Comparison of demographic information of parents of premature babies

Variables		Experimental group (*n* = 42)		Control group (*n* = 40)		p-value
		***N (%)***	Mean ± SD	***N (%)***	Mean ± SD	
Mother’s age (year)	Less than 20	11(29.7)		10 (27.8)		*0.269
	20–29	10 (27)		17 (47.2)		
	30–39	10 (27)		7 (19.4)		
	≥ 40	6 (16.3)		2 (5.6)		
Mother’s age (year)			27.27 ± 9.02		25.11 ± 7.42	
Father’s age(year)	20–29	15 (40.5)		18 (50)		*0.202
	30–39	13 (35.1)		12 (33.3)		
	≥ 40	9 (24.3)		6 (16.7)		
Father’s age(year)			33 ± 8.91		30.44 ± 8.01	
Mother’s education	Elementary - Guidance	17 (45.9)		15 (41.7)		**0.167
	High school- Diploma	12 (32.4)		18(50)		
	University	8 (21.6)		3 (8.3)		
Father’s education	Elementary - guidance	1 (2.7)		2 (5.6)		***0.546
	High school - diploma	13 (35.1)		16 (44.4)		
	University	23 (62.2)		18 (50)		
Mother’s Occupation	Housewife	31 (83.1)		26 (72.2)		**0.233
	Employed	6 (16.2)		10 (27.8)		
Father’s Occupation	Manual worker	4 (10.8)		7 (19.4)		***0.683
	Employee	11 (29.7)		10 (27.8)		
	Free	20 (54.1)		16 (44.4)		
	Unemployed	2 (5.4)		3 (8.3)		
Insurance	Has it	27 (73)		23 (63.9)		**0.404
	Does not have	10 (27)		13(36.1)		
Economic status	Optimal	11 (29.7)		7 (19.4)		**0.509
	Relatively favorable	18 (48.6)		18 (50)		
	Undesirable	8 (21.6)		11 (30.6)		
Number of children	1	18 (48.6)		18 (50)		***0.843
	2	14 (37.8)		15 (41.7)		
	3	5 (13.5)		3 (8.3)		

* Independent t-test

** Chi-square test

*** Fisher’s exact test

In addition, there was no significant difference between the two groups in terms of clinical variables of premature babies (birth type, baby gender, baby weight, Apgar, and age) (Table 2).

**Table 2:** Comparison of clinical characteristics of premature babies

Variables		Experimental group (*n* = 42)		Control group (*n* = 40)		p-value
		**N (%)**	Mean ± SD	**N (%)**	Mean ± SD	
Type of birth	Cesarean section	21 (56.8)		25 (69.4)		**0.262
	*NVD	16 (43.2)		11 (30.4)		
Infant’s sex	Girl	19 (51.4)		21 (58.3)		**0.549
	Boy	18 (48.6)		15 (41.7)		
Baby’s weight (grams)	< 1400	8 (21.6)		11 (30.6)		***0.144
	2400 − 1400	19 (51.4)		16 (44.4)		
	> 2400	10 (27)		9 (25)		
Baby’s weight (grams)			20,215 ± 480.36		1851.94 ± 521.65	
Apgar	7	3 (8.1)		5 (13.9)		****0.607
	8	11 (29.7)		8 (22.2)		
	9	23 (62.2)		23 (63.9)		
Age (Week)	< 30	5 (13.5)		7 (19.4)		**0.712
	30–35	18 (48.6)		18 (50)		
	> 35	14 (37.8)		11 (30.6)		

* Normal vaginal delivery

** Chi-square test

*** Independent t-test

**** Fisher’s exact test

The results show that the stress of 40.5% of the fathers in the experimental group was at an average level and 10.8% had a low level of stress, while in the control group, none of them had low stress and the majority, i.e., 88.9%, had very high stress (Table 3).

**Table 3:** Comparison of the mean stress and its subscales after the intervention in the fathers of premature babies of experimental and control groups

Stress and its subscales	Experimental group (*n* = 37)	Control group (*n* = 36)	Mean Difference	*p-value	Effect Size (95% CI)
	Mean ± SD	Mean ± SD			
NICU environment	11.89 ± 7.08	17.47 ± 5.76	5.58	< 0.001	0.86(0.37, 1.33)
Appearance and behavior of the baby and the special treatments that are given to the baby	33.56 ± 12.76	56.83 ± 13.41	23.27	< 0.001	1.78(1.22, 2.30)
Parent Parent-child relationship and parenting roles	18.86 ± 10.44	35.44 ± 10.3	16.58	< 0.001	1.60(1.06, 2.11)
Total stress	64.32 ± 24.12	109.75 ± 22.24	45.43	< 0.001	1.96(1.38, 2.49)

*Independent t-test Cohen Effect Size = 0.2 small, 0.5 medium, 0.8 large

**Table 4:** Comparison of the mean state anxiety of experimental and control before and after intervention

State anxiety	Experimental group (*n* = 37)	Control group (*n* = 36)	Mean difference	*p-value	Effect Size (95% CI)
	Mean ± SD	Mean ± SD			
Before intervention	50.70 ± 11.08	55.33 ± 11.01	4.63	0.077	-
After intervention	43.21 ± 9.68	54.22 ± 10.39	11.01	< 0.001	0.86 (0.37, 1.33)
* p-value *	< 0.001	0.001		-	

*Independent t-test

**Paired-sample t-test Cohen Effect Size = 0.2 small, 0.5 medium, 0.8 large

The within-group comparison showed that there was a significant difference in state anxiety in the control group before and after the intervention (Table 4). Table 5 shows that the changes in state anxiety in the experimental group were significantly higher than in the control group(*p* < 0.001).

**Table 5:** Comparison of the difference in the mean state anxiety between experimental and control groups

State anxiety	Experimental group (*n* = 37)	Control group (*n* = 36)	*p-value	95% CI (L/H)
	Mean ± SD	Mean ± SD		
State anxiety	−7.48 ± 4.85	−1.11 ± 1.76	< 0.001	(4.66/8.08)

*Independent t-test

## Discussion

This study investigated the effect of the support program on fathers’ stress and anxiety during their initial encounter with their premature baby.

The findings revealed a significant reduction in mean stress scores, as measured by the Parental Stressor Scale: NICU (PSS: NICU), and its subscales, alongside improved state anxiety scores on the State-Trait Anxiety Inventory (STAI) in the experimental group compared to the control group. Notably, most fathers in the intervention group reported moderate stress levels, whereas fathers in the control group predominantly experienced high stress, highlighting the program’s effectiveness in reducing paternal distress during this critical early stage of parent–infant interaction.

Our findings are consistent with those of Shahkalahi et al. [29], who reported reductions in overall parental stress and in specific subscale scores such as infant appearance/behavior, parent-child relationship, following a support intervention. However, their study found no significant change in NICU environment stress, possibly because participants’ prior exposure to the unit 2–4 days after admission, whereas fathers in our study were engaged within 12 h of birth. This earlier timing may have amplified the intervention’s impact, as providing support during peak anxiety is particularly effective [30]. In contrast, Matricardi et al. [19] observed that joint observation and infant massage significantly reduced maternal but not paternal stress, unlike our results. This discrepancy may reflect differences in intervention focus, as their program was designed for both parents rather than specifically tailored to fathers. Similarly, Zahid Pasha et al. [31] reported that virtual education reduced stress but not anxiety, whereas our face-to-face approach effectively lowered both outcomes.

Busse et al. [15] and Noergaard et al. [32] support our results by highlighting that early that early father–infant contact and a father-inclusive NICU environment enhance attachment and decrease stress. A systematic review also reinforces these findings, indicating that “hug care” is both safe and beneficial for fathers and infants, suggesting a bidirectional mechanism of stress reduction [33]. These findings suggest that incorporating father-specific, face-to-face support programs into NICU protocols could enhance paternal well-being and promote family-centered care, particularly in regions with high preterm birth rates. The intervention’s success in the Iranian context, where familial structures often emphasize paternal responsibility and support, indicates its potential applicability in other cultural settings that similarly value fathers’caregiving roles. However, generalizability may be influenced by cultural variations in gender roles and levels of paternal access to the NICU. For instance, in contexts with less paternal NICU involvement (e.g., some Western contexts) or different healthcare infrastructures, adaptation of the program may be needed. Evidence from a Greek study [34] and a related survey [35] indicate that younger fathers or those in resource-limited settings may benefit more, necessitating tailored approaches. Future studies should explore multi-center trials across diverse cultural and socioeconomic settings to validate these findings. Additionally, the low dropout rate (< 10%) supports feasibility, but addressing barriers to participation (e.g., language, education) could broaden reach.

The intervention, which combined individualized informational and emotional support with active paternal participation in preterm infant care, effectively reduced fathers’ stress and anxiety within three days of birth. These results suggest that early, structured support can meaningfully alleviate acute psychological distress among fathers in the NICU. Nevertheless, the short-term evaluation period constrains understanding of the intervention’s long-term impact. Components such as guided imagery to foster hope and facilitated soothing interactions with the infant may have sustained benefits for paternal mental health and parent-infant bonding. Strengthening fathers’ confidence and resilience could potentially reduce the risk of long-term postpartum depression or anxiety, while early emotional and physical engagement with the infant may reinforce attachment and foster positive family dynamics over time.

Future longitudinal research is warranted to explore these sustained outcomes. Follow-up assessments at 6, 12, and 24 months postpartum could evaluate enduring effects on paternal mental health and family relationships. In addition, integrating qualitative methodologies, such as semi-structured interviews, would capture the evolving emotional trajectories and perceptions of fathers as caregivers. Such insights could contribute to a more comprehensive understanding of the intervention’s mechanisms, informing the refinement of holistic, family-centered care models in NICU settings.

This study’s findings, demonstrating significant reductions in paternal stress and anxiety following a targeted neonatal intensive care unit (NICU) intervention, gain depth when interpreted through established psychological and sociological frameworks. According to Lazarus and Folkman’s stress and coping theory, as applied in O’Brien et al. (2013), elucidates how fathers appraise NICU-related stressors, such as infant vulnerability and altered parental roles, and employ coping strategies, including emotional support and active involvement, to mitigate distress [36]. The intervention’s emphasis on resilience aligns with this framework, promoting adaptive coping mechanisms to alleviate anxiety. From a sociological perspective, role theory, as discussed by Noergaard et al. (2018), helps explain how traditional masculine norms, particularly those prevalent in Iranian society, may discourage emotional expression and amplify paternal stress. By encouraging direct caregiving and open emotional involvement, the intervention challenged conventional paternal role boundaries, thereby promoting more balanced and inclusive family dynamics [18]. Furthermore, Bowlby’s attachment theory, as applied in Fegran et al. (2008), underscores the importance of early parent–infant interaction in shaping secure attachment patterns. Facilitating early contact and soothing behaviors between fathers and their preterm infants may not only strengthen emotional bonds but also buffer long-term psychological strain and enhance paternal identity [37]. Integrating these theoretical perspectives provides a comprehensive understanding of how the intervention operates across emotional, cognitive, and social dimensions. This theoretical synthesis not only enriches the interpretation of the current findings but also offers a conceptual foundation for developing future family-centered, father-inclusive NICU interventions aimed at optimizing both parental and infant well-being.

### Research limitations

This study is subject to several limitations that warrant consideration.

-  The variable utilization of educational booklets and DVDs by fathers in the experimental group may have influenced outcomes, despite the researcher’s emphasis on their use and the provision of a dedicated phone number to address related queries. This inconsistency in engagement could introduce variability in the intervention’s effectiveness, though it reflects real-world challenges in participant adherence.
-  The existing body of comparative literature remains limited, as few studies have specifically focused on fathers of preterm infants. Most prior research has predominantly examined mothers or combined parental data without gender-specific analysis, thereby constraining the depth of contextual comparison and interpretation of the present findings.
- The reliance on self-reported stress and anxiety measures, which are subject to social desirability and cultural biases, particularly among men in societies where stoicism and emotional restraint are valued, such as in Iran. This tendency may have led to underreporting of psychological distress and bonding challenges, potentially underestimating the true impact of the intervention. Future studies should therefore incorporate qualitative methods, including interviews or focus groups, to capture richer, more nuanced emotional data. Such triangulation would deepen understanding of paternal psychological experiences, strengthen the validity of findings, and advance the evidence base for father-inclusive, family-centered NICU care.
- The single-center design of this study, conducted at Shahid Motahari Hospital, Urmia, Iran, may also limit generalizability due to contextual factors such as cultural expectations of paternal roles and the organizational structure of Iranian teaching hospitals. However, the intervention’s simplicity and adaptability, requiring minimal resources and comprising visual imagery, educational materials, and emotional support, enhance its potential for adaptation across diverse healthcare systems and cultural contexts. The intervention can be tailored to align with local norms, such as varying expectations of paternal involvement, with educational content customized to reflect culturally relevant depictions of fatherhood. Critically, its efficacy depends on a compassionate and knowledgeable facilitator with expertise in preterm infant care, ensuring its applicability in resource-limited settings.
- It is important to acknowledge that the control group in this study received standard NICU support, limited to routine explanations of their infant’s condition by nursing staff, which may have exaggerated the intervention’s observed effects due to the minimal support provided. This minimal control condition, while consistent with typical NICU practices in the study setting, may not fully delineate the specific contributions of the intervention’s components, such as emotional support and active paternal involvement. Employing an active control group in future research, for example, by providing general educational materials without personalized emotional support, could better isolate the unique effects of the intervention and thereby enhance the rigor and interpretability of results.
- The lack of blinding for participants and NICU staff, inherent to the intervention’s nature (active support vs. standard care), introduces potential performance bias, despite blinding of outcome assessors to mitigate assessment bias. Future studies should address these limitations through extended follow-ups, multi-center designs, and enhanced blinding protocols where feasible.

## Conclusion

This study demonstrates that the stress and anxiety experienced by fathers of preterm infants were significantly ameliorated through a support program compared to the control group. These findings underscore the efficacy of informational support, coupled with active participation in infant interaction, in reducing paternal stress and anxiety during neonatal intensive care unit (NICU) admissions. Healthcare systems must prioritize father-specific needs by integrating evidence-based, family-centered care models into routine practice. Specific policy recommendations include mandating the implementation of structured, face-to-face support programs within the first 12 h of NICU admission, training NICU staff in father engagement techniques, and providing accessible educational resources (e.g., illustrated booklets and DVDs) with follow-up support. Such changes can foster inclusive care environments and improve paternal and familial outcomes. Nevertheless, the generalizability of these results warrants further validation through multi-center, randomized controlled trials across diverse cultural and healthcare contexts.

## Supplementary Information

Supplementary material 1.

## Data Availability

The datasets used and analyzed during the current study are available from the corresponding author on reasonable request.

## Abbreviations

STAI: State-trait anxiety inventory
PSS: NICU: Stressor scale: neonatal intensive care unit
IRCT: Iranian registry of clinical trials
