# Supplementary figures and images for: “Effect of a support program on fathers’ stress and anxiety during initial NICU encounter with premature infants: a randomized clinical trial”
Source: Reprod Health. 2025 Dec 29;22:261. doi: 10.1186/s12978-025-02204-w (PMC12751724; doi:10.1186/s12978-025-02204-w)

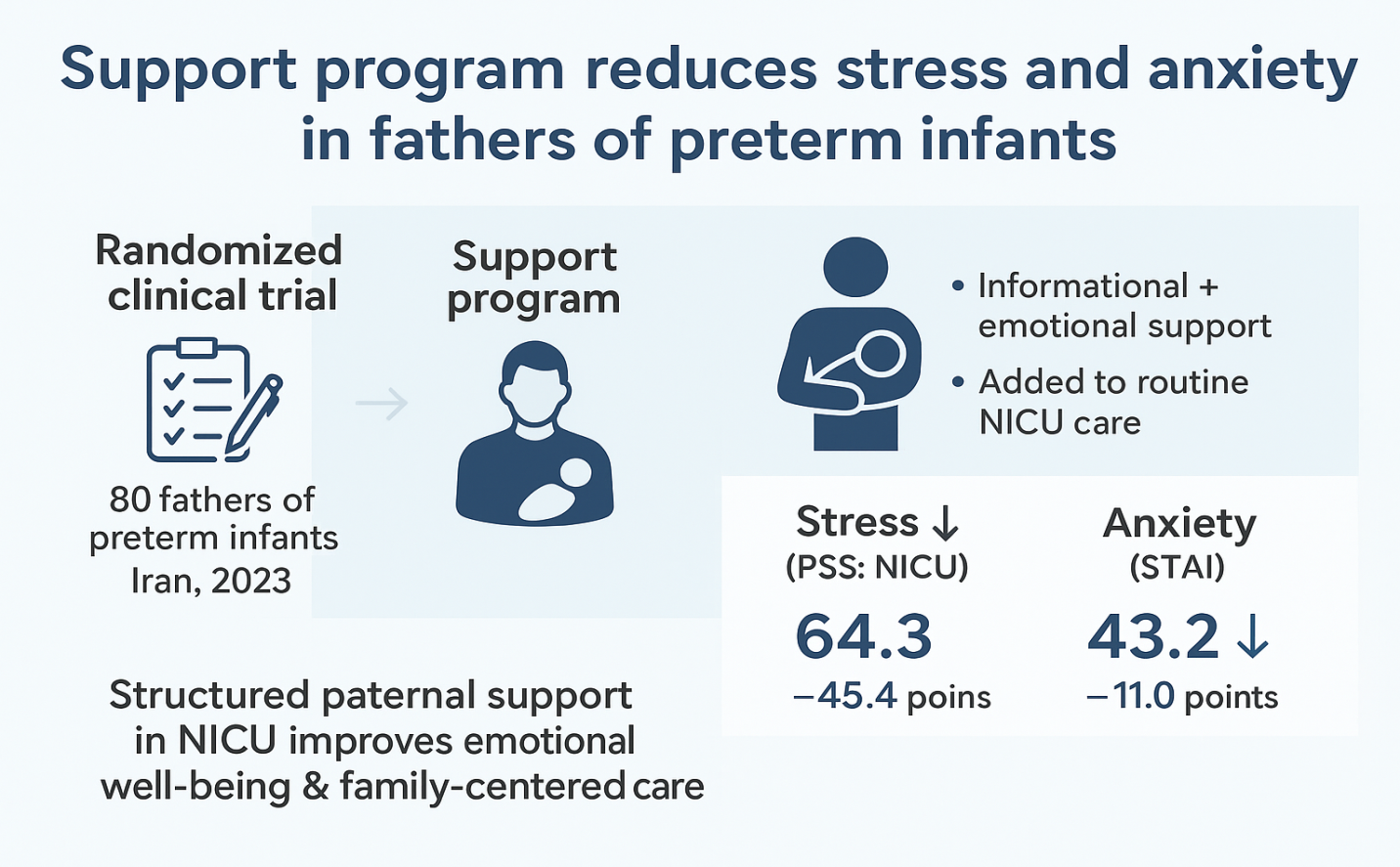

Supplement: Supplementary file 1 — Supplementary material 1. [file 12978_2025_2204_MOESM1_ESM.docx]
